# The Significance of Properly Reporting Turnover Frequency in Electrocatalysis Research

**DOI:** 10.1002/anie.202110352

**Published:** 2021-09-15

**Authors:** Sengeni Anantharaj, Pitchiah Esakki Karthik, Suguru Noda

**Affiliations:** ^1^ Department of Applied Chemistry School of Advanced Science and Engineering Waseda University 3-4-1 Okubo, Shinjuku-ku Tokyo 169-8555 Japan; ^2^ Waseda Research Institute for Science and Engineering Waseda University 3-4-1 Okubo, Shinjuku-ku Tokyo 169-8555 Japan; ^3^ Department of Chemical Engineering Hanyang University 222 Wangsimni ro, Seongdong-gu Seoul 04763 Republic of Korea

**Keywords:** CO_2_ reduction, electrocatalysis, N_2_ reduction, turnover frequency, water splitting

## Abstract

For decades, turnover frequency (TOF) has served as an accurate descriptor of the intrinsic activity of a catalyst, including those in electrocatalytic reactions involving both fuel generation and fuel consumption. Unfortunately, in most of the recent reports in this area, TOF is often not properly reported or not reported at all, in contrast to the overpotentials at a benchmarking current density. The current density is significant in determining the apparent activity, but it is affected by catalyst‐centric parasitic reactions, electrolyte‐centric competing reactions, and capacitance. Luckily, a properly calculated TOF can precisely give the intrinsic activity free from these phenomena in electrocatalysis. In this Viewpoint we ask: 1) What makes the commonly used activity markers unsuitable for intrinsic activity determination? 2) How can TOF reflect the intrinsic activity? 3) Why is TOF still underused in electrocatalysis? 4) What methods are used in TOF determination? and 5) What is essential in the more accurate calculation of TOF? Finally, the significance of normalizing TOF by Faradaic efficiency (FE) is stressed and we give our views on the development of universal analytical tools to determine the exact number of active sites and real surface area for all kinds of materials.

## Graphical abstract

Turnover frequency (TOF) serves as an accurate descriptor of the intrinsic activity of a catalyst. However, if it is reported at all, it has not been accurately determined in most of the recent studies on electrocatalytic reactions involving both fuel generation and fuel consumption. This Viewpoint stresses the significance of realistic TOF data in the development of new electrocatalysts.

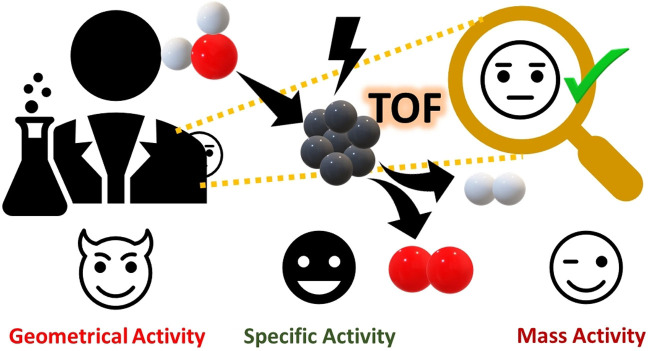

## Introduction

The electrochemical conversion of small molecules into fuels and value‐added products in electrolysers and electrical energy in fuel cells is catalyzed by materials of appropriate energies of interaction in order to avoid a huge loss in efficiency.[^1^, ^2^] The electrosplitting of H_2_O, CO_2_, N_2_, N_2_H_4_, NH_3_, BH_4_^−^, etc.[^3^, ^4^, ^5^, ^6^, ^7^, ^8^] is categorized as fuel formation and the electrolytic consumption of H_2_, alcohols, and simple sugars for electric energy generation in fuel cells is categor*ize*d as fuel consumption.[^9^, ^10^] In both fuel‐forming and fuel‐consuming electrocatalytic conversion of small molecules, one or more products are formed at a certain rate and this rate is what determines how efficient a catalyst is for the reaction of interest.[^11^, ^12^, ^13^, ^14^, ^15^]

Conventionally, in all electrocatalysis studies, a set of activity markers are used to benchmark the performance of a catalyst or a set of catalysts. Among them, the most frequently used activity markers are overpotential (*η*) and exchange current density (*j*_0_).[^16^, ^17^, ^18^, ^19^, ^20^, ^21^] Overpotential is the measure in volts that describes the additional electromotive force required by the catalyst to begin the reaction from its equilibrium potential (mathematically, *η*=*E*−*E* ^0^). In the determination of overpotential, several practices have been followed, such as reporting the onset overpotential, half‐wave potential, or a potential at a fixed current density.[^11^, ^12^] The onset potential has been emphasized in the oxygen reduction reaction (ORR),[^22^, ^23^, ^24^] two‐electron water oxidation reaction (2 e^−^ WOR) for H_2_O_2_ electrosynthesis,[^25^, ^26^, ^27^, ^28^, ^29^] CO_2_ reduction reaction (CRR),[^7^, ^8^] and nitrogen reduction reaction (NRR).[^5^, ^30^, ^31^] On the other hand, the overpotential at 10 mA cm^−2^ has been given significance in the oxygen evolution reaction (OER) and hydrogen evolution reaction (HER), though it is not an ideal practice.[^11^, ^12^]

All these overpotentials are almost exclusively determined from the current responses normalized by the geometrical surface area of the electrode, which is never the same as the real surface area of the interface because none of the electrodes used in the abovementioned electrocatalytic reactions has a perfectly smooth and planar surface in practice.[^12^, ^32^] In fact, most of the recently reported nanostructured materials modified/supported electrodes tend to have a significantly higher surface area than what is projected by the geometry of the substrate electrode.[^33^, ^34^, ^35^, ^36^] This results in a huge uncertainty in the activity determined. Hence, these markers actually reflect the apparent activity and not the intrinsic activity.

Though apparent activity is important in a practical context, one cannot go ahead with electrode design and structure without having any idea of the intrinsic activities of the catalysts. Therefore, researchers have begun reporting other markers that reflect the intrinsic activity better than the overpotentials used currently. These alternative markers include the specific activity and mass activity in which the current response is normalized by the electrochemical surface area (ECSA) and the mass of the loaded catalyst, respectively.[^16^, ^37^] However, these two ways of normalizing the current response and determining the overpotential thereof have their own limitations and uncertainties, making them poor intrinsic activity markers; this will be discussed later in this Viewpoint.

The *j*_0_ introduced earlier is another activity marker which is simply the current that flows across the catalytic interface at the reversible or equilibrium potential of the reaction under study.[^17^, ^20^, ^38^] This is generally obtained from the extrapolation of the linear portion of the Tafel line. However, because of the limitations in using potentiodynamic polarization curves for Tafel analysis and the effect of uncompensated resistance (*R*_u_) that exists in all electrical circuits, the precise calculation of *j*_0_ has been an ongoing problem.^[20]^ Despite all these ambiguities, the overpotential determined with a current response normalized using the geometrical area of the electrode and the *j*_0_ determined from a Tafel line, which is in turn extracted from potentiodynamic polarization curves, have been used as the primary markers without a broad understanding that these markers only reflect the apparent activity and not the intrinsic activity.^[11]^ The main reason why such this practice prevails despite having a higher degree of unreliability for intrinsic activity determination is that the amount of product formed at the catalytic interface is directly proportional to the amount of charge passed as per the laws of Faraday. Hence, the current response obtained is assumed to reflect the intrinsic activity without consideration of the effects of the real surface area, series resistance, capacitive behavior of the interface, lowered coulombic efficiency, etc. This practice is inadvertently based on the wrong belief that *j*_0_ reflects the intrinsic activity.

In this context, a better way to assess the intrinsic activity of any catalyst would be to determine its turnover frequency (TOF), which is simply the measure of the ratio of product formed per unit time and the amount of catalyst used (precisely, the exact number of catalytic sites participating).[^12^, ^39^] Though most researchers are aware of it and have been using the TOF?, the amount of product formed in electrocatalysis is not directly determined to calculate the TOF, especially in water electrosplitting.^[11]^ Instead, the current density normalized by the geometrical area of the electrode is typically used. This current density is affected by parasitic and other competing reactions as stated above. Hence, immediate attention should be paid to amend the practices followed in TOF determination at this stage. Undoubtedly, TOF could be a better intrinsic activity marker but the issues related to its determination prevent this. Hence, in this Viewpoint we justify why TOF should be preferred to characterize intrinsic activities over other widely used markers that reflect apparent activity in electrocatalysis and how TOF can be obtained with improved accuracy.

## Widely Used Activity Markers in Electrocatalysis

### An Overview of Overpotential and Exchange Current Density

When it comes to the electrocatalytic conversion of small molecules, the very first thing that researchers look into is the overpotential required by the electrocatalyst used, which apparently represents the efficiency of the whole electrode assembly rather than the intrinsic activity. As mentioned earlier, there are several conventions and conditions to determine overpotentials, depending on the reaction under study. For example, in electrocatalytic water splitting, it is the overpotential at 10 mA cm^−2^ which was adopted in analogy with the standards set for solar to fuel energy conversion devices, which in fact was recently the subject of a serious debate.[^40^, ^41^, ^42^] Current consensus among leading researchers in the field is that a potential at which 1 mA cm^−2^ is achieved can be used to benchmark the water‐splitting electrocatalysts.^[2]^ However, for catalysts that have dominant parasitic reactions, such as the self‐oxidation of the catalyst and significant capacitive behavior, neither 1 nor 10 mA cm^−2^ can be set as benchmarking conditions.^[43]^ This is often encountered with nanostructured electrocatalysts supported on carbon cloth (CC) and foam‐type electrodes and porous materials having high surface area.^[37]^ Also, catalysts grown on foil‐type electrodes with huge loadings are no exception. In such cases, researchers use either the backward sweep of the cyclic voltammogram (CV) or select a higher current density such as 50 or 100 mA cm^−2^, whichever is appropriate.^[11]^ This precludes a fair and reasonable comparison of activities reported in different studies. Hence, a standard unification of activity markers is desired.

Similarly, the onset overpotential has been given more significance in CRR, ORR, NRR, MOR, and 2 e^−^ WOR.[^5^, ^25^, ^44^] In some of these reactions (especially in ORR), the half‐wave potential is also of interest to researchers. In these complex multistep reactions, the possibility of obtaining different products is very high. Hence, the Faradaic efficiency (FE) is also given equal importance beside overpotentials.[^25^, ^45^] Another activity marker is *j*_0_, which is conventionally obtained by the extrapolation of the linear region of the Tafel line to the reversible potential of the reaction studied. Just like the geometrical area normalized and FE‐neglected current density used for overpotential determination, *j*_0_ is also affected by geometrical area normalization and apparently does not reflect the intrinsic activity of the catalyst. Nonetheless, it is not as widely used as overpotential as an activity marker these days.

### Why Overpotentials and *j*_0_ Cannot Reflect Intrinsic Activity

In general, the current response acquired with any of the commonly used DC electroanalytical techniques is normalized by the geometrical area of the electrode, which is never the same as the truly projected area, as most of the electrocatalysts studied these days are nanostructured and tend to have a higher surface area with a very high roughness factor.^[12]^ Hence, geometrical area normalization exaggerates the activity of almost every electrocatalyst reported. To overcome this issue, specific activity and mass activity are frequently used.^[11]^ Of these two, mass activity represents the current response normalized by the loaded amount of the catalyst with the unit A g^−1^. Though it appears rational when compared to activity normalized by the geometrical area, the issue with this is that not all the active sites in the loaded catalyst are exposed to the electrolyte and involved in the reaction. Only the active sites that are directly exposed to the electrolyte solution are responsible for the observed current response. Besides, the leaching of catalyst during the reaction lowers the actual mass of the catalyst. This means that normalizing the current response for the entire reaction time by the loading measured before the reaction can be quite misleading. Hence, normalizing the current response by the amount of catalyst loaded is not an ideal way even for reflecting the apparent activity. This can cause serious deviations in results when samples of the same amount of the same material but having different particle size and shape (so that the exposed area is different) are studied under identical conditions. The specific activity, on the other hand, is a more precise way to assess the (intrinsic) activity of an electrocatalyst, in which the current response is normalized by the real surface area of the catalyst.^[16]^ However, the precise determination of active/real surface area of an electrocatalyst under study is very difficult.^[46]^

There are a few reliable techniques such as hydrogen underpotential deposition (HUPD), catalyst's redox peak integration, CO and Cu striping, but they are material‐specific and cannot be used universally for all kinds of electrocatalysts. Besides, for most of the materials used these days, there is no universal and precise method for calculating the exact number of active sites and real surface area including the double‐layer capacitance (*C*_dl_) method. In the *C*_dl_ method, the obtained *C*_dl_ is divided by the specific capacitance (*C*_s_) of the material studied, which is not usually determined as a part of the same experiment but taken from literature sources.^[18]^ This increases the odds of severe reproducibility issues. Moreover, these *C*_dl_ values are not constant and tend to vary significantly with time (of exposure to electrolyte), electrolyte composition, and catalyst lifetime.

Another practice that is occasionally followed in specific activity determination is the use of Brunauer–Emmett–Teller (BET) isotherms for calculating the specific surface area (SSA) of a material.^[41]^ Unfortunately, this method has several serious limitations. The SSA calculated by this method basically corresponds to the area of gas (N_2_ in most of the cases) adsorption and desorption sites. Not all of these gas adsorption and desorption sites are also electrochemically active. Moreover, this method is suitable only for powder catalysts and not for supported electrocatalysts that may otherwise require scratching or sonicating which may significantly alter the real surface area. During these processes, the catalyst particles may also agglomerate and settle on top of each other, masking a significant number of active sites. In such a case, using BET‐determined SSA to normalize the current response obtained with a surface‐modified electrode almost always underreports the activity. Hence, overpotentials calculated with any (geometrical activity, specific activity, and mass activity) of the abovementioned current density may never represent the intrinsic activity but the apparent activity of the whole interface (i.e., substrate | catalyst | electrolyte). This is true for *j*_0_ as well because the Tafel lines are extracted mostly from the LSV/CV/CA responses constructed using areal activity, specific activity, and mass activity.[^20^, ^41^]

In general, FE is another important phenomenon that can significantly influence the activities determined by these methods.^[16]^ Even when one can obtain the real surface area of an electrocatalyst for normalizing the current response, the determined activity cannot be the true one (except for catalysts with 100 % FE, which is rare among electrocatalytic energy conversion reactions and small‐molecule activations) if the FE of the catalyst is unknown. Hence, espousing a reliable and a straightforward method that can reflect the intrinsic activity of an electrocatalyst such as TOF with FE normalization is desirable at this stage.

## TOF: A Better Way of Showing the Intrinsic Activity

TOF is a simple and a straightforward intrinsic activity marker that can project how efficient a catalyst is for the reaction of interest, as it is the measure of the amount of product formed or reactant consumed per unit time for the given amount of catalyst.[^47^, ^48^] This applies to electrocatalysts as well. The following discussion is centered on the methods used for TOF calculations in electrocatalysis (mainly water splitting), existing challenges and ambiguities, appropriate methods, and plausible universalization of finding active sites for all types of catalysts.

### Methods of Calculating TOF

**Figure 1:**
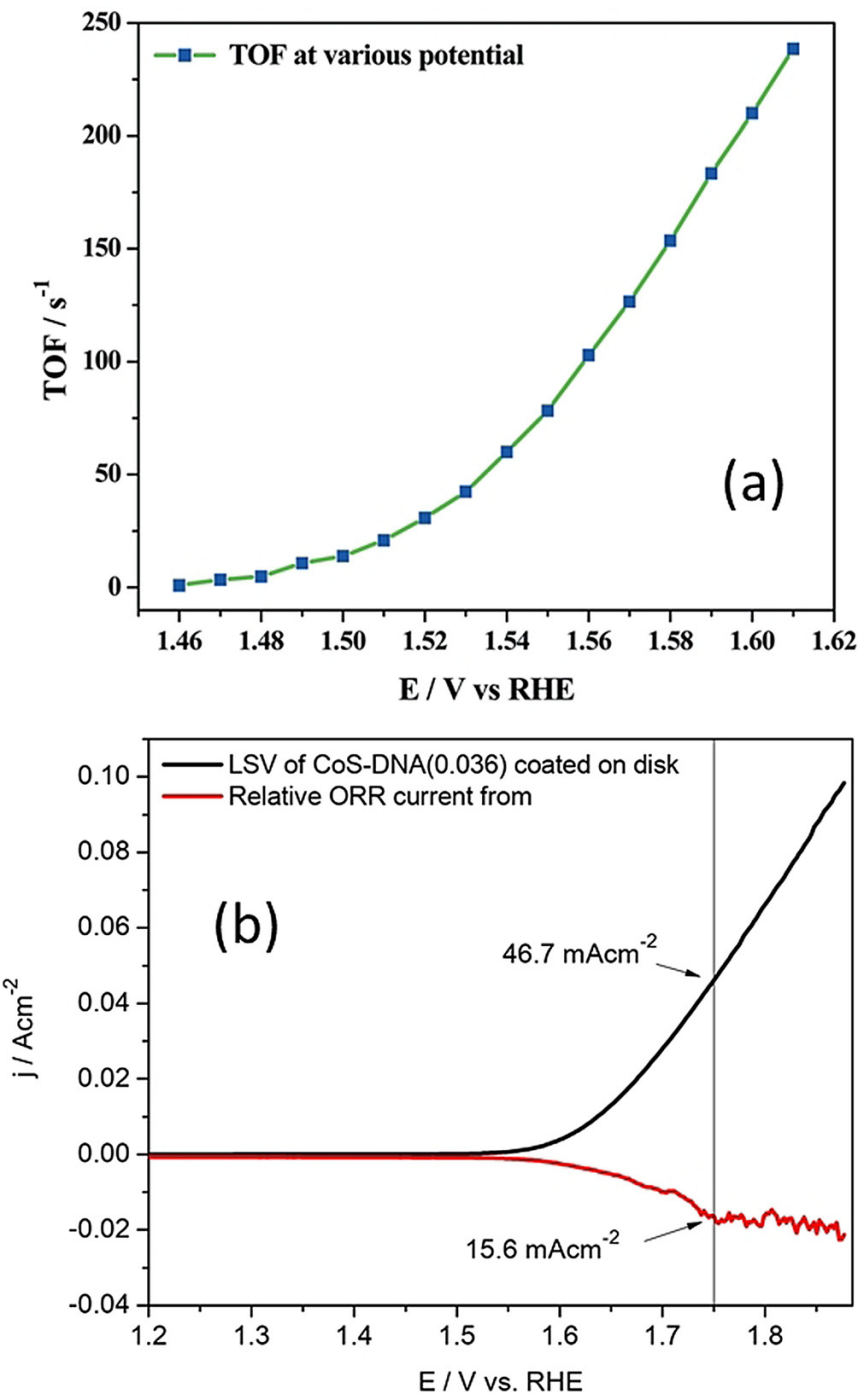
a) Plot of TOF as a function of potential. Reproduced from ref. [49]. Copyright 2017, The Royal Society of Chemistry. b) RRDE current–potential response of CoS‐DNA OER catalyst in 1.0 M KOH. Reproduced from ref. [50]. Copyright 2017, American Chemical Society.

In electrocatalytic water splitting, both half‐cell reactions (OER and HER) are being intensively studied and every day new electrocatalysts are reported. Unfortunately, most of these recent studies do not include TOF data and report just the apparent activity markers mentioned above. In contrast to classical heterogeneous catalysis, the amount of product formed (H_2_ in HER or O_2_ in OER) is not measured in electrocatalysis. Instead, the current density is used along with other necessary parameters, and hence, TOF values are always reported as a function of overpotential (Figure 1 a).^[49]^ Many equations are used for TOF calculation in electrocatalysis; Equation 1 is the most commonly used,

(1) $$\mathrm{TOF}=j\times N{}_{A}/\left(F\times n\times \Gamma \right)$$

where *j, N*_A_ *, F, n*, and *Γ* represent current density, the Avogadro constant, the Faraday constant, the number of electrons transferred to generate one molecule of the product, and the surface concentration or exact number of active sites catalyzing the reaction (m^−2^), respectively. The value of *n* is 2 for HER and 4 for OER. A few researchers also use variations of Equation (1), [Eqs. (2) and (3)], where *i* and *A* in Equation (2) stand for the current and area of the electrode and *x* in Equation (3) stands for the number of moles of active sites available for the catalysis, respectively.

(2) $$\mathrm{TOF}=i\times N{}_{A}/\left(A\times F\times n\times \Gamma \right)$$

(3) TOF=j/(x×F×n)

When Equation (3) is used for calculating the TOF of HER, the value of *n* is 2, whereas, for OER, it is 4. This is simply the number of electrons transferred when a molecule of H_2_ or O_2_ is formed.

For OER, an indirect and handy method is also used which is the determination of TOF of OER by concurrent ORR at the ring electrode using a rotating ring disk electrode (RRDE) assembly.^[50]^ In this method, the catalyst‐modified disk electrode is subjected to a potential ramp over a desired potential window within which OER occurs significantly while the ring electrode is kept at a constant cathodic potential sufficient for reducing the O_2_ evolved from the disk electrode (Figure 1 b). In this case, Equation (4) is used for TOF calculation.

(4) $$\mathrm{TOF}=i{}_{R}/\left(A\times F\times n\times N{}_{\mathrm{CL}}\times \Gamma \right)$$

Here, the terms *i*_R_, and *N*_CL_ stand for ring current and the collection efficiency of RRDE used, respectively. Though manufacturers of RRDEs provide the standard *N*_CL_ value, it tends to change significantly upon use. Hence, it is always better to determine the *N*_CL_ of the RRDE used frequently with standard redox probes such as ferro–ferri redox couple. Details on this can be found in our earlier work.^[49]^ This method can also be used for HER by setting a constant anodic potential at the ring electrode which is sufficient enough to cause the concurrent electrooxidation of H_2_ evolved from the disk electrode, the potential of which is swept in the cathodic region.

In this case, the value of *n* is 2, as H_2_ electrooxidation is a two‐electron reaction. However, one should be vigilant in setting the anodic potential carefully as the HER and HOR are not separated well by a larger overpotential window like OER and ORR. In such a scenario, complications such as a competitive HUPD could derail the objective of the whole study.

There is also another approach which does not involve current, Faraday constant (*F*), and the variations thereof. In this method, the quantity of H_2_ or O_2_ evolved is determined directly by means other than recording the current, for example by conventional water displacement with an inverted graded cylinder and GC‐MS. The water displacement method is straightforward yet it requires more time as the catalyst must generate a sufficient volume of gas that can be determined precisely with the naked eye. Although this is a straightforward method, there are complications such as catalyst degradation during the study and high chances of human error during the measurement. On the other hand, GC‐MS with a H_2_ sensor has been in use for the determination of FE in HER.^[51]^ This also can be used for TOF calculation, provided the exact number of active sites is known.

Though the previously discussed methods seem to be impeccable in determining the TOF and revealing the intrinsic activity of an electrocatalyst, there are many other challenges and ambiguities in currently followed practices of calculating TOF.

### Challenges in the Current Approaches to Calculating TOF

Although TOF is a straightforward intrinsic activity marker, its calculation in electrocatalytic studies has always been unwittingly flawed due to the complications in determining the exact number of active sites participating in the catalysis. In addition, the use of geometrical area normalized current density further drives the accuracy of TOF determination away from the reality. This section elaborates on the most commonly used methods of determining the exact number of active sites and/or real surface area with examples and lists their advantages and disadvantages for better understanding. The exact number of active sites is to be exploited for calculating the TOF using the current normalized by the real surface area.

### Methods of Determining the Real Surface Area

Several electrochemical methods are used to determine the exact number of active sites, or the real surface area, of which HUPD,^[52]^ Cu underpotential deposition (CuUPD),^[53]^ Pb underpotential deposition (PbUPD),^[54]^ CO striping,[^46^, ^55^, ^56^, ^57^] and redox peak integration[^58^, ^59^, ^60^, ^61^] are the most noteworthy. Unfortunately, these methods are very material‐specific and cannot be applied universally for all electrocatalysts.

#### Underpotential Deposition (UPD)

In electrochemistry, the concept of reversible or equilibrium potential is well‐known and it is defined as the potential at which an ion deposits reductively on the surface of the same with zero‐valent atoms (i.e., deposition of M^*n*+^ on M^0^). In contrast, when a metal ion is deposited on the surface of another metal/surface with a high work function (*φ*), the reductive deposition occurs at lower cathodic potentials than its reversible or equilibrium potential; this anodic shift in the deposition potential is the chemical potential of the surface on which underpotential deposition takes place. This phenomenon is widely known as underpotential deposition (UPD).[^62^, ^63^] Many metallic surfaces display this characteristic deposition of different ions. Familiar examples include the underpotential deposition of H on Pt, Cu on Au, and Pb on Cu.

**Figure 2:**
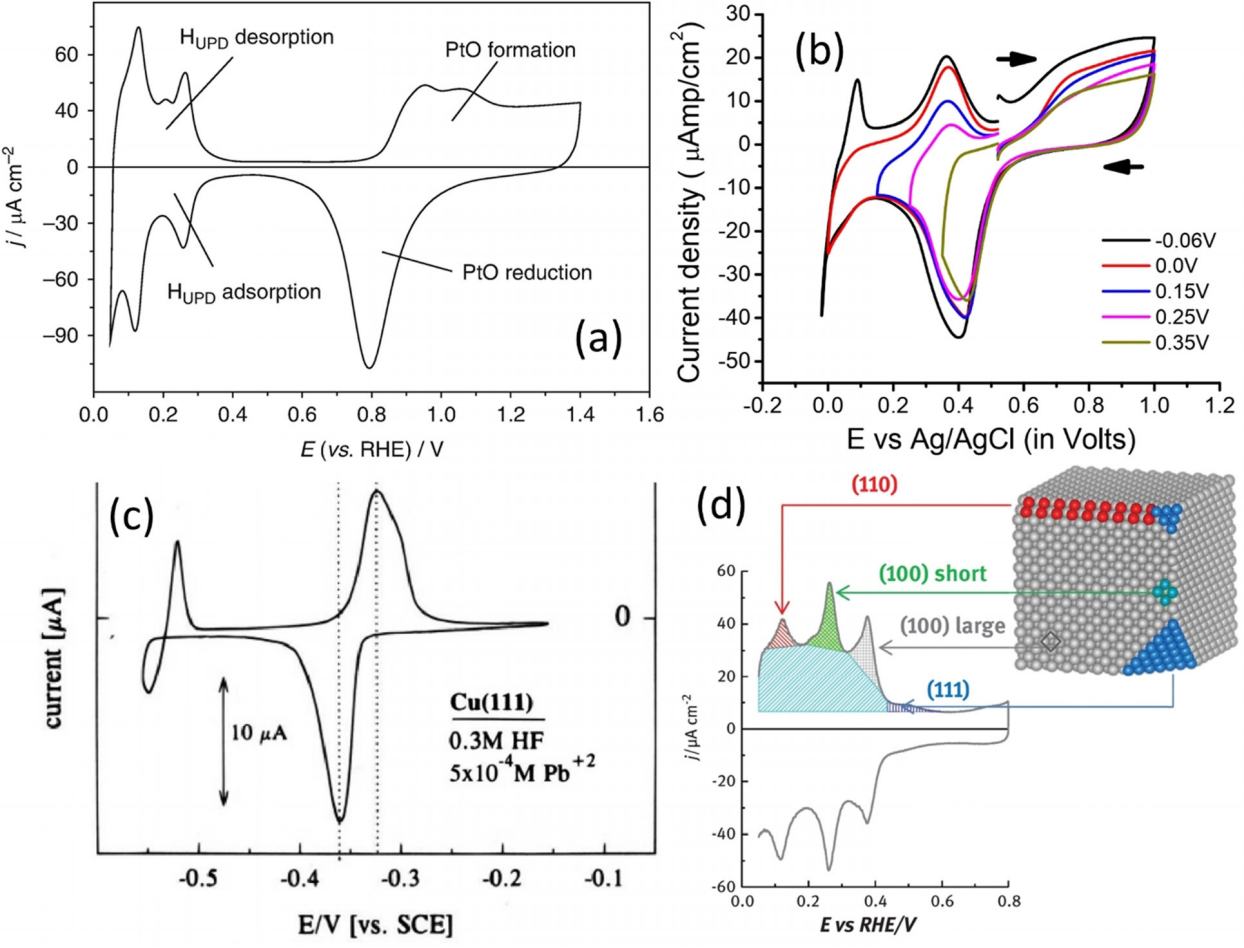
a) Cyclic voltammogram of a polycrystalline Pt electrode in 0.5 M H_2_SO_4_ acquired with 10 mV s^−1^ showing HUPD peaks. Reproduced from ref. [67]. Copyright 2020, Springer. b) Cyclic voltammograms showing Cu UPD peaks on a Pd surface with cathodically increasing cut‐off potentials in a solution containing 0.500, 0.010, and 0.001 M of NaClO_4_, HClO_4_, and Cu(ClO_4_)_2_, respectively. Reproduced from ref. [68]. Copyright 2015, American Chemical Society. c) Cyclic voltammogram showing Pb UPD on a Cu(111) surface in 0.3 M HF containing 0.0005 M of Pb^2+^ ions. Reproduced from ref. [54]. Copyright 1995, American Chemical Society. d) Cyclic voltammogram features that would be observed for HUPD on different planes on a Pt electrode in 0.5 M H_2_SO_4_. Reproduced from ref. [69]. Copyright 2017, Elsevier.

Many other possible substrate–ion combinations and a detailed account on underpotential deposition have been given by Mayet and co‐workers recently.^[53]^ In electrocatalytic water splitting and CO_2_ electroreduction, the two most studied catalytic surfaces are Pt and Cu, respectively.[^64^, ^65^, ^66^] The exact electrochemical surface area of Pt can be determined by HUPD^[67]^ and of Pd by CuUPD^[68]^ (Figure 2 a,b), while that of Cu can be determined by PbUPD (Figure 2 c).^[54]^ This electrochemical surface area can then be used to normalize the current response to obtain the specific activity. However, in order to calculate the exact number of active sites or the number of moles of active sites for TOF calculation, the crystallographic information of the metallic surface catalyzing the reaction of interest must be known because the charge integrated under the UPD peak is highly dependent on the faceting of the catalytic surface.^[67]^ For example, the HUPD charge of Pt for planes (100) and (111) is 200 and 230 μC cm^−2^, respectively, whereas the polycrystalline surface will have a charge of 210 μC cm^−2^ in pH 0. Moreover, the difference in faceting notably influences the shape of the HUPD peaks as well (Figure 2 d).^[69]^ Unfortunately, nanostructured electrocatalysts used these days are mostly polycrystalline with a single or a couple of dominant facets; in these cases, it would be significantly incorrect to assume such fixed values of UPD charges calculated for certain planes to calculate the real surface area.

#### Stripping

Stripping is another efficient way of determining the real surface area of a metallic surface in which a strongly chemisorbed monolayer of a particular species (can be an ion or charged/neutral ligands capable of forming complexes with the metallic surface under study through back‐bonding) on a metallic surface is electrochemically oxidized (i.e., the monolayer is stripped off the surface by electrochemical oxidation).^[57]^

**Figure 3:**
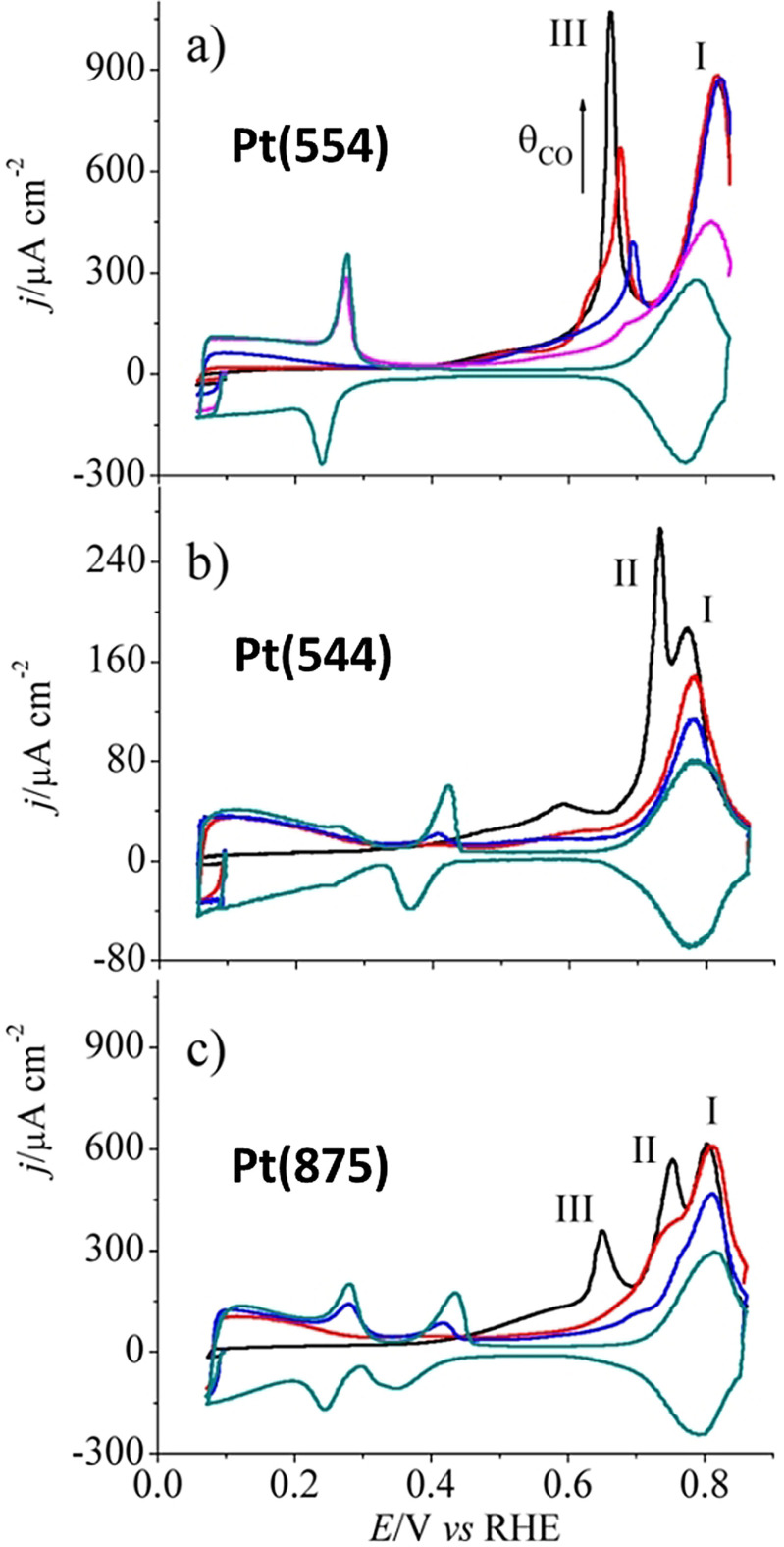
a–c) CO stripping voltammetry of Pt electrodes faceted with (554), (544), and (875) planes after CO sub‐monolayer growth effected at 0.100 V vs. RHE. The black line represents the stripping of CO with 100 % coverage while the red and blue lines correspond to the stripping of CO of intermediate coverage. The dark cyan lines are the blank responses. Reproduced from ref. [71]. Copyright 2013, American Chemical Society.

From the quantity of charge passed across the interface during stripping and the number of electrons transferred in the reaction, one can easily quantify the amount of the species that was chemisorbed on the metallic surface. This value is an indirect measure of the real surface area of the metallic surface under study. A very well‐known example is the CO stripping of Pt and Pd.^[70]^ In methanol and other small‐carbon‐fuel‐based fuel cell electrocatalysis, oxidation following a multistep reaction produces CO as a key intermediate, which is a strongly back‐bonding low‐spin ligand known to form metal carbonyl complexes. In metal carbonyls, the metal center is almost always in its native oxidation state. Pt is a widely used electrocatalyst in fuel cells, including those that use alcohols and other CO‐producing fuels. Hence, CO poisoning (formation of Pt carbonyls) has been a major issue in this area. Recent studies with non‐Pt catalysts, and catalysts that are alloyed with other CO‐eliminating metals such as Ru, has restored the hope of commercializing this technology.^[10]^ Though CO poisoning has been a curse in the field of fuel cells, it has become a handy and reliable tool for studying the real surface area of several metals including Pt and Pd. A simple CV sweep in CO‐purged electrolyte reveals the clear oxidation peak of CO monolayer stripping (Figure 3 a–c) from which the real surface area can be calculated and used for specific activity determination.^[71]^ Just like HUPD, CO stripping is also dependent on the crystallographic facets and can vary in intensity depending on the coverage as shown in Figure 3 a–c.

Similarly, despite being accurate ways of calculating the real surface area, UPD and stripping techniques cannot be used for all electrocatalysts. Only the metal surfaces that exhibit UPD towards a particular species in solution and those that form electrochemically completely oxidizable monolayers of a ligand (such as CO) can be studied. Besides, these two methods reveal the real area in cm^2^, which can only be directly used specific activity determination. For TOF calculations, these real surface area values must be converted into the exact number of active sites based on the crystallographic data of the material used; this is far from trivial. Further complications arise when the metal surface used is polycrystalline or amorphous or combination thereof, for which little or no standard data on the relationship between the real surface area and UPD/CO stripping charge are available. Hence, an alternative method of determining the exact number of active sites is essential, albeit UPD and stripping can give the real surface area.

#### Redox‐Peak Integration

This is the easiest approach and an efficient way of finding out the number of active catalytic sites. The major limitation is that the redox‐peak integration method is applicable to only monometallic catalysts that possess a redox couple within the potential window of the electrolytic process catalyzed on its surface such as Ni, Co, Cu, Fe, Mn, Pt, Ir, and Ru.[^58^, ^60^, ^61^, ^72^] Electrocatalytic water oxidation is a reaction that involves bond‐forming and ‐breaking intermediate steps, requiring the catalytic sites to undergo a complete cycle of oxidation and reduction. Hence, it is safe to assume that the number of metal sites undergoing oxidation prior to water oxidation is the exact number of sites participating in the reaction. Transition metal‐based OER electrocatalysts do undergo distinct self‐oxidation to a higher valence state which is usually responsible for the oxidative splitting of water. Hence, if the number of electrons transferred in the oxidation/reduction of single site is known, one can easily calculate the total number of electrons transferred based on the charge under the peak, which in turn can be used to calculate the exact number of catalytic sites. This can be then used in both TOF and specific activity determinations.

**Figure 4:**
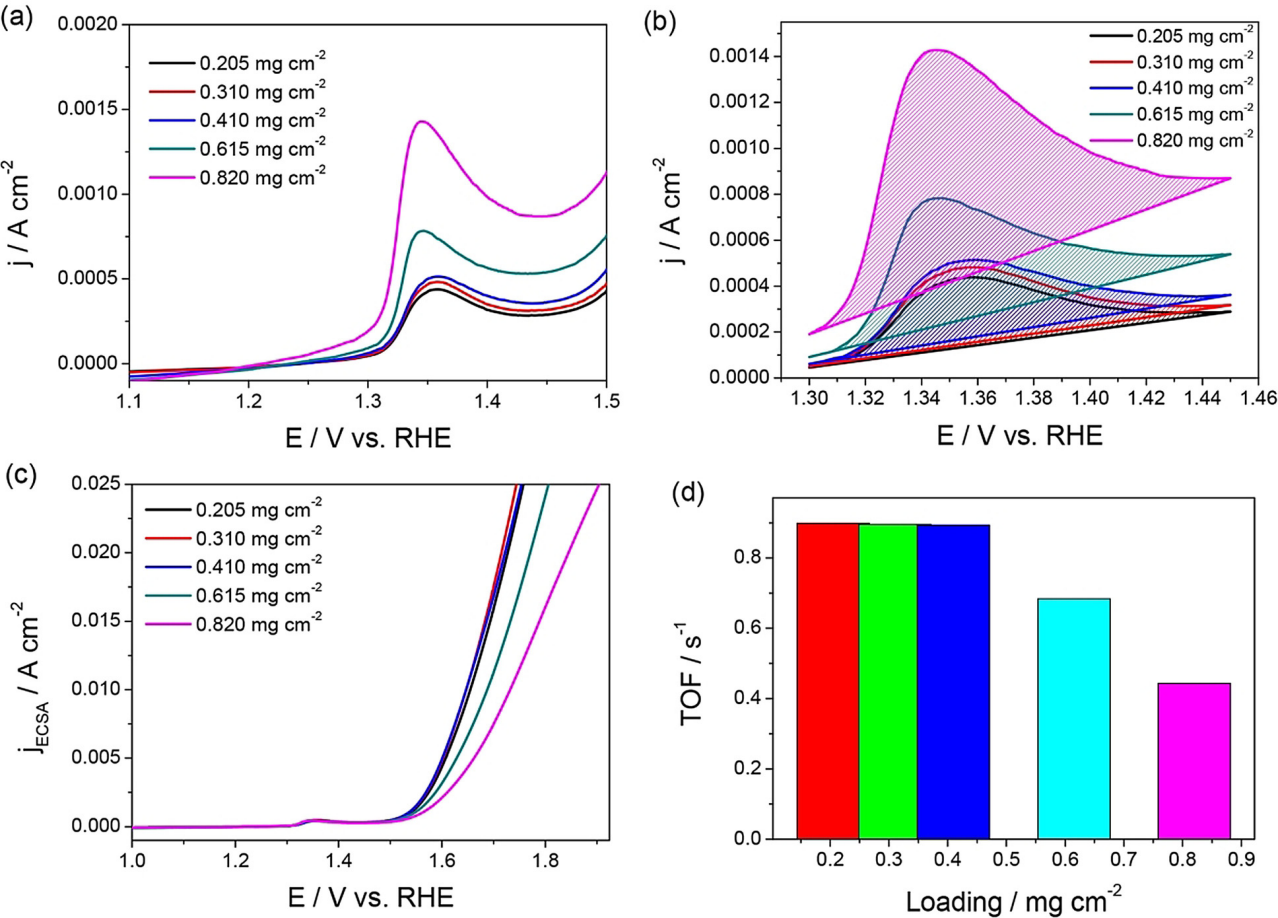
a) LSVs of NiO OER electrocatalysts showing a distinctive oxidation peak which increases with increasing loading, which is ascribed to the increase in the number of accessible sites. b) The isolated oxidation peaks of the same LSVs used for charge integration and the calculation of the number of active sites. c,d) Specific activity and TOF determined using the calculated number of sites from the charge integrated from the oxidation peaks shown in (b). Reproduced from ref. [12]. Copyright 2018, American Chemical Society.

**Figure 5:**
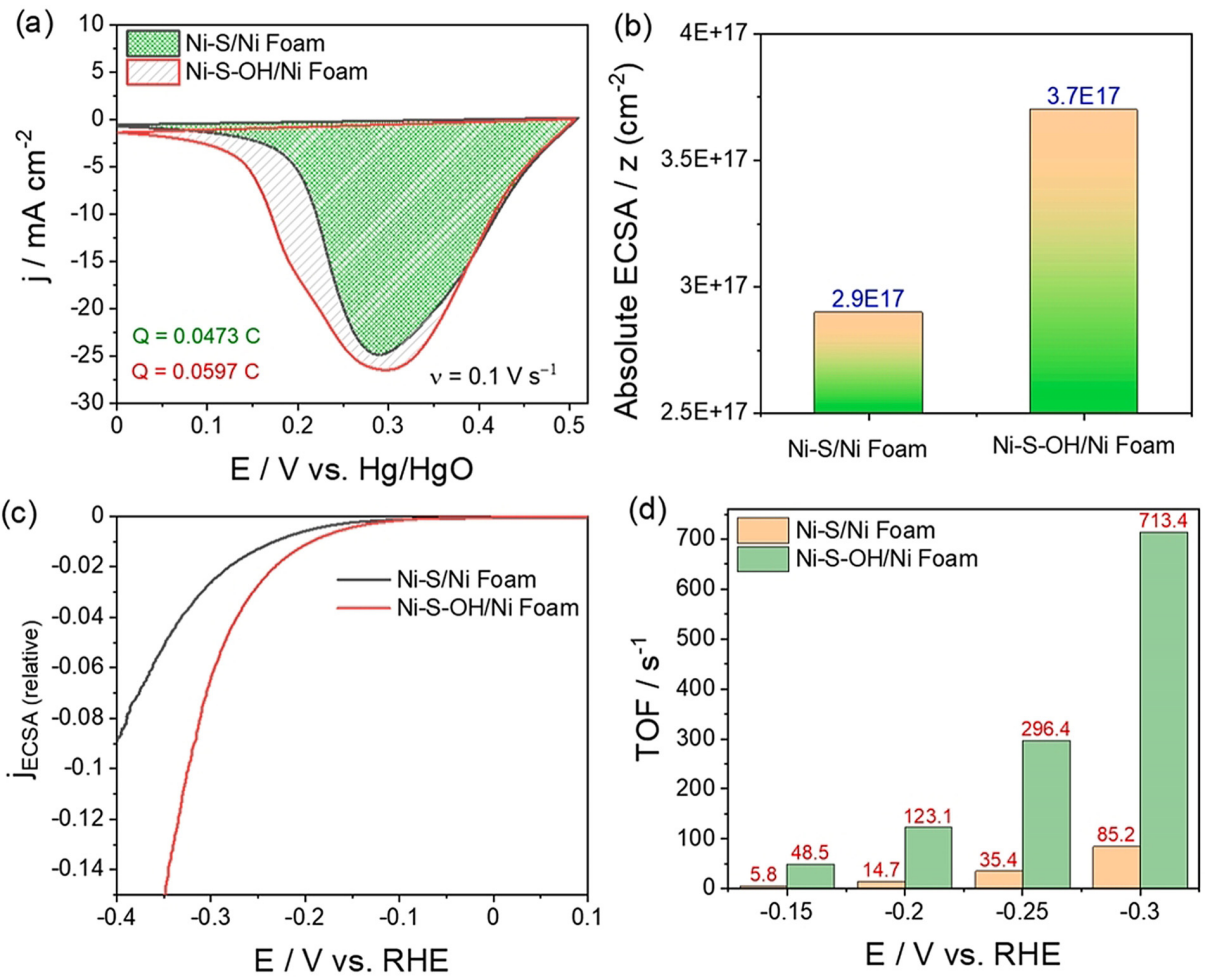
a) The reduction peak of Ni^3+^ to Ni^2+^ used to calculate the number active sites in NiS and hydroxylated NiS. b) Respective absolute ECSA obtained from the integrated charge. c,d) Specific activity and TOF determined for HER catalyzed by the same materials. Reproduced from ref. [61]. Copyright 2021, Elsevier.

An example is Ni‐based OER electrocatalysts (Figure 4 a–d) in which Ni lies in the 2+ state before its further oxidation to Ni^3+^ in nickel oxyhydroxide, which is responsible for OER.^[12]^ Since it is a single‐electron‐transfer reaction, the calculation of the exact number of active sites can be made straight away. Such a number determined from these anodic oxidation peaks can also be used to determine specific activities and TOF for the reductive splitting of water (i.e., HER) catalyzed by the same catalyst in the same environment (Figure 5 a–d).^[61]^

**Figure 6:**
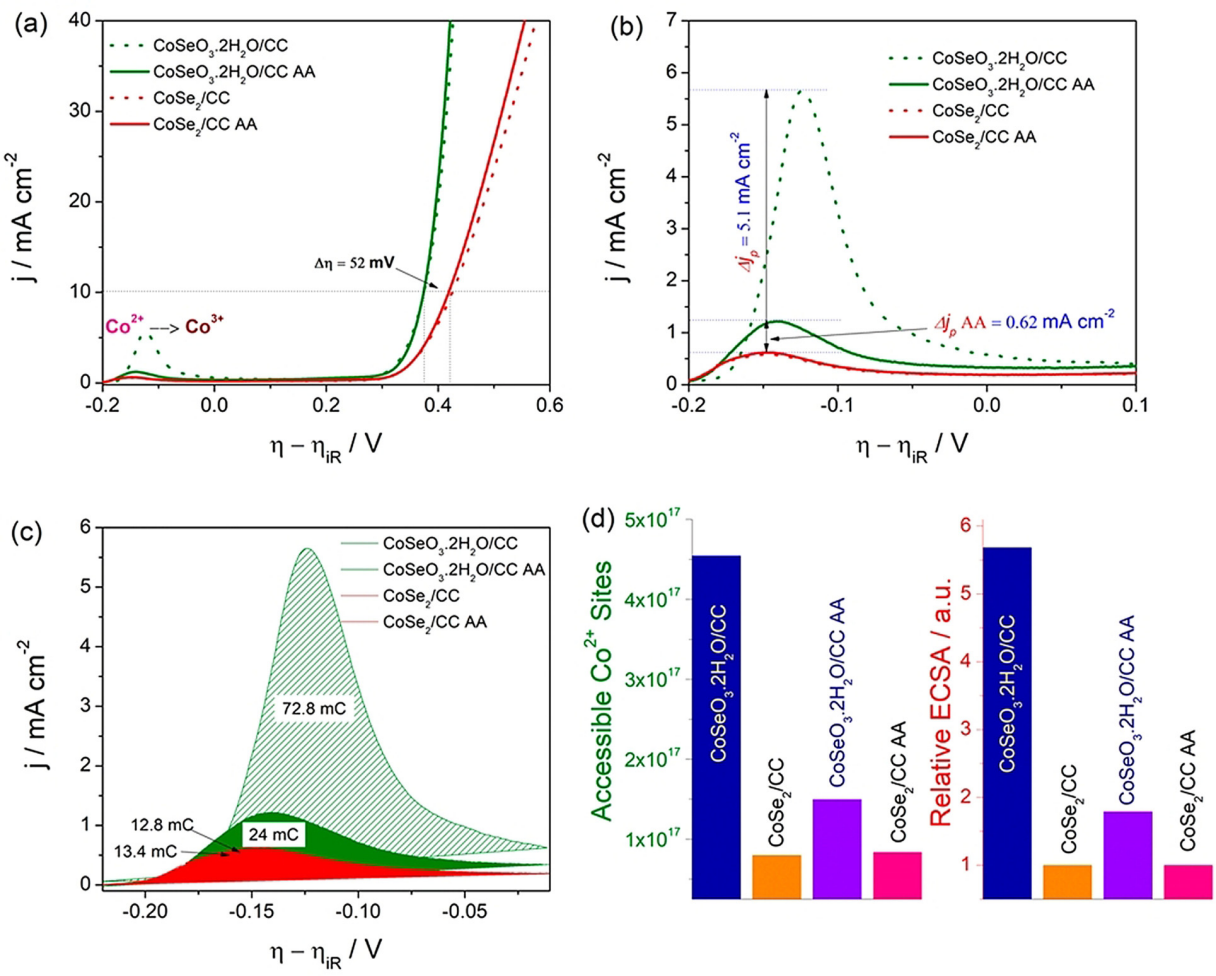
a) LSVs of selenide and selenite of Co^2+^ showing the difference in the oxidation peak current densities before and after activation (i.e., between the first run and after 100^th^ run). b,c) Enlarged version of the same oxidation peaks showing the difference in peak current and integrated charge under the peaks. d) Exact number of accessible sites and the relative ECSA calculated using the integrated charges of first and 100^th^ run. AA: after activation. Reproduced from ref. [58]. Copyright 2020, American Chemical Society.

Other monometallic catalysts such as the oxides/hydroxides and non‐oxide/hydroxides of Co, Cu, Ir, and Ru can also be examined in the same manner.^[73]^ However, in the case of catalysts that show distinctive differences in their oxidation peaks between the first run and the consecutive runs, it is always better to use the oxidation peak obtained in the first run for charge integration and the determination of the exact number of accessible sites. An example for this is Co‐based catalysts in which the oxidation of Co^2+^ to Co^3+^ in the first cycle is dominant and occurs at a lower potential and the oxidation peak of Co^2+^ (resulting from the partial reduction of Co^3+^ formed in the first anodic sweep) in the consecutive cycles is suppressed in current density and shifted anodically (Figure 6 a–d).^[58]^ This is mainly because a significant part of the Co^3+^ oxidized in the first cycle stays at this state and is involved in a redox reaction with Co^4+^, which was later proven to be the major contributor in the Co‐based catalysts for water electrooxidation.[^74^, ^75^, ^76^] In addition, one can also use the backward sweep and integrate the charge under the reduction peak. However, it is still indispensable to stick with the first cycle for catalysts that show distinct redox behaviors such as Co‐based catalysts. Though this method appears to be accurate, it suffers from other limitations such as the addition of capacitive current to the oxidation peak and the irreversibility or quasi‐reversibility of the redox couple, which affect the charge under the oxidation peak at every run. Hence, using the oxidation peak of the first cycle seems to be appropriate. Besides, serious complications may arise when the oxidation of all the accessible sites is not complete in the first cycle. This is the case where the catalysts are porous, nanostructured, and hierarchical assemblies in nature that take a considerable time for complete wetting so that they do not expose all the accessible sites within the time scale of the first run. To overcome this, catalyst‐modified or supported electrodes can be kept at OCP for a sufficiently long time before their LSV/CV are recorded. Despite all of the above, this method cannot be used for bi‐ and multimetallic catalysts whose redox couples merge as a result of alloying. Hence, like UPD and stripping techniques, this is also not a universal approach although it is a more precise way to calculate the exact number of active/accessible sites. However, UPD/stripping and redox‐peak integration can be used complementarily to find out ECSA and the number of active sites for both noble and non‐noble monometallic electrocatalysts, which is clearly an advantage that is not available for bimetallic or metal‐free electrocatalysts.

#### Double‐Layer Capacitance (*C*_dl_)

**Figure 7:**
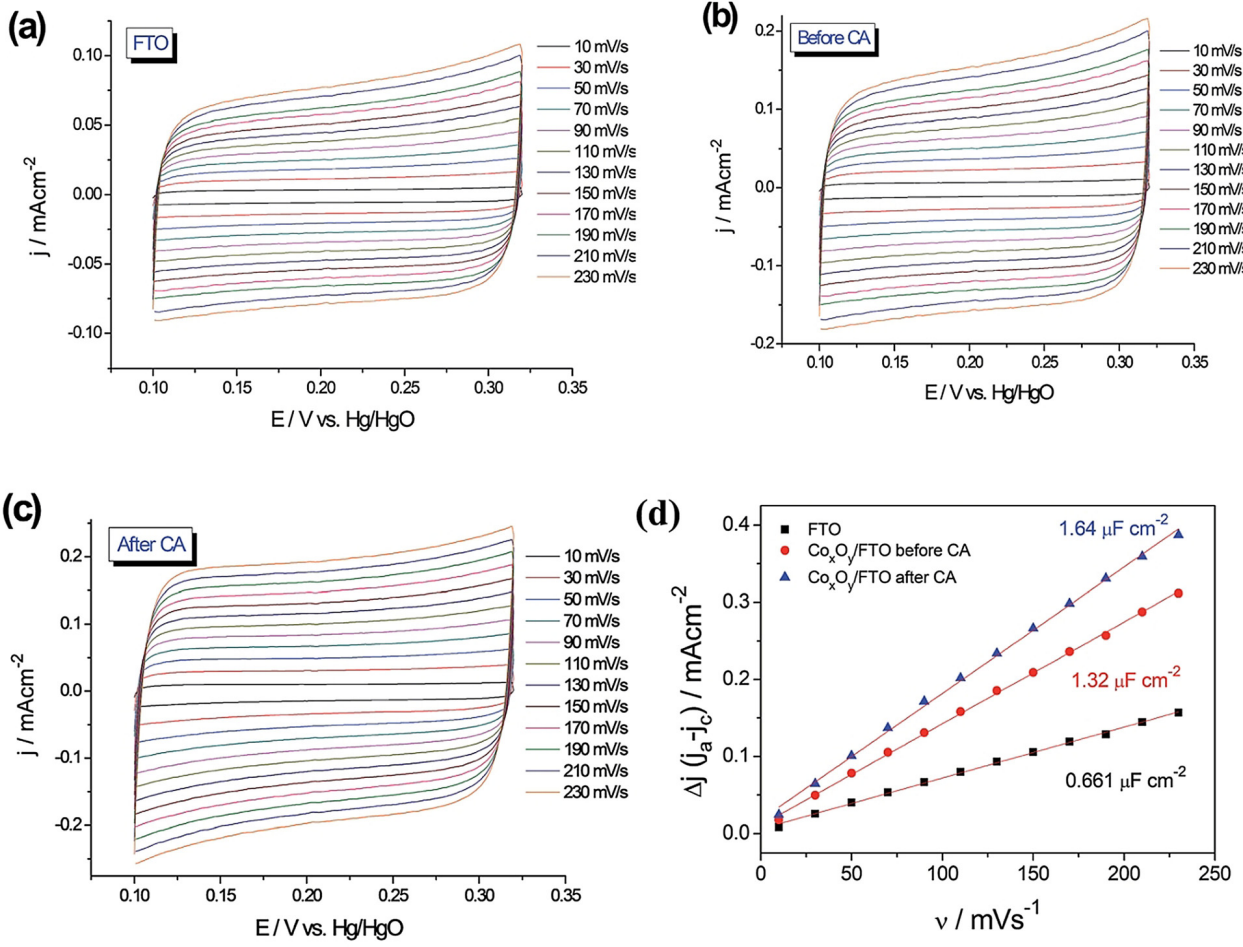
a–c) CV responses of fluorine‐doped tin oxide (FTO), Co_*x*_O_*y*_‐coated (by pulsed laser deposition) FTO before CA, and after CA, respectively. d) The plot of double‐layer charging current densities against the scan rates shows an increase in 2 *C*_dl_, indicating an increase in the electrochemical surface area. Reproduced from ref. [78]. Copyright 2017, The Royal Society of Chemistry.

Non‐Faradaic currents that result from the adsorption and desorption of ions in the electrolyte on the surface of the electrode are also used to calculate the real surface area of an electrocatalyst.^[18]^ The main advantage of this method is that it is universally applicable to all electrocatalysts from simple metals to oxides/hydroxides, chalcogenides, and pnictides of metals. However, the accuracy and reproducibility of this method is very poor when compared to the methods (UPD, striping, and redox peak integration) discussed above. It is because, each time a new electrode is made by modifying the same material via conventional catalyst ink modification, the area exposed to the electrolyte changes and this will always result in different *C*_dl_ values. Besides, the solid‐state properties and the wettability of the catalyst will also significantly affect the *C*_dl_ measured. In principle, for the electrode under investigation, CVs of a narrow potential window (150 to 300 mV) in the non‐Faradaic region closer to that of the desired electrocatalytic reaction are obtained with increasing scan rate.^[11]^ Either charging or discharging current can be plotted against the scan rate to get the *C*_dl_ value from the slope of the resulting linear line. For electrodes and interfaces that are complex in topography, charging and discharging currents are not always equal. Hence, to ensure a better credibility, researchers plot both charging and discharging currents (obtained by subtracting the non‐Faradaic current of the cathodic sweep from that of the anodic sweep and expressed as Δ*j* or *j*_a_−*j*_c_) against the scan rates to get 2 *C*_dl_ from which *C*_dl_ can be obtained.^[77]^ To calculate the electrochemical surface area (note that it is not the same as the electrocatalytically active/accessible surface area/sites), this *C*_dl_ is divided by the specific capacitance (*C*_s_) of the material that makes up the electrode. Most of the time, *C*_s_ values are adapted from another study where the same material was used, which, however, cannot be the same when it has different surface properties and environments (electrolytes, pH, etc.).^[48]^ Hence, a relative ECSA calculated by assuming the *C*_dl_ of the substrate electrode with a smooth surface and of area 1 cm^2^ is equivalent to the capacitance of the material with 1 cm^2^ area, which again is not a rational assumption as the charging and discharging characteristics of the substrate and the material coated on the substrate can never be the same. Because of these many disadvantages, the *C*_dl_ method despite being a universal way of calculating electrochemical surface area is not suitable for determining the exact number of active sites. Moreover, researchers are hesitant towards using the *C*_dl_ method to calculate the electrocatalytically active surface area or justify catalytic activity because there is no guarantee that a given material has the same number of adsorption/desorption sites and catalytically active sites. However, it has still been widely used to calculate the roughness factor and to justify the electrocatalytic activity differences that arise mainly due to changes in electrochemical surface area and the loading. An example is given Figure 7 a–d, where the increase in the electrochemical surface area is revealed just by following the *C*_dl_ values of substrate and the pristine catalyst film coated substrate before and after activation.^[78]^

Nonetheless, this method still cannot be used to calculate the exact number of active sites. Among the four approaches elaborated here, the first three are very accurate but not universal, whereas the *C*_dl_ method is universal but not accurate. This urges for the development of methods that can be both accurate and universally applicable in the future.

### Using Redox Probes to Determine Real/Electrochemical Surface Area

In addition to the methods discussed earlier, there are several other methods which can also be used to determine the true surface area or the electrochemical surface area for the normalization of current responses that result in specific activity. However, they may not reveal the exact number of active sites participating in the reaction being catalyzed. In fact, without knowing the exact number of active sites participating in the catalysis (this can fairly be converted into area in cm^2^ if the size, shape, lattice parameters, and density are known), it is literally impossible to calculate a more accurate TOF. Making assumptions such as 100 % participation of all the sites in the loaded catalyst could potentially demean the purpose of TOF determination for the reasons discussed earlier. Nonetheless, methods that use redox probes can come in handy in determining real/electrochemical surface area, and thereby they can increase the degree of accuracy in TOF determination when used to find the specific activity. When lattice parameters of the material are known, these can also be used to find out the exact number of active sites as well.

There are several redox probes with perfect reversibility and other required Nernstian characteristics that can be used to find out the electrochemical surface area of any electrode surface. Familiar ones are hexacyanoferrate(II) & hexacyanoferrate(III), hexamine ruthenium(III), ferro–ferri oxalate, and hydroquinone–quinone.[^79^, ^80^, ^81^, ^82^, ^83^] The main issue with these redox probes is that they are pH dependent, have short solution lifetimes (ferro–ferri cyanide forms Prussian blue after a certain time in acid), and cannot be used in the same electrolyte in which the actual electrocatalytic reaction is carried out. For example, the ferro–ferri cyanide complex is used in 0.5 M H_2_SO_4_, which is handy for HER catalysts performed in 0.5 M H_2_SO_4_ but this data cannot be used for OER and HER catalysts in highly alkaline solutions, where the hydrolysis of ferro–ferri complex occurs in no time. In such instances, the area determined with these probes in a different electrolyte can be significantly different from the actual value in an electrolyte where the desired reaction is catalyzed. Also, there are other issues such as the nature of the interaction of these probes with the electrode surface and the nature of electron transfer mechanism they follow. A few redox probes stay right beside the inner Helmholtz plane without any direct contact and perform electron transfer via the outer‐sphere mechanism obeying Marcus theory.^[84]^ Hexamine ruthenium(III) is a well‐known redox probe of this kind.^[85]^ On the other hand, a few redox probes specifically adsorb onto the electrode surface (i.e., penetrating the inner Helmholtz plane) and perform electron transfer via the inner‐sphere mechanism, which also includes the destruction and reconstruction of the coordination sphere. Ferro–ferri cyanide and ferro–ferri oxalate are the best‐known examples of this kind.[^79^, ^80^] These characteristics of a redox probe can have a profound effect on electrochemical surface area determination. Detailed accounts on various redox probes and their applicability can be found elsewhere.[^79^, ^80^, ^81^, ^82^, ^86^]

### Thoughts on Futuristic Universal Ways (Material‐Independent) of Determining the Exact Number of Electroactive Sites

All the methods discussed above have their own practical shortcomings and are material‐specific. This has been the most immovable hurdle to determining the most accurate TOF for all kinds of electrocatalysts. In this section, we propose how the recently evolved in situ and operando spectroelectrochemical, electrochemical microscopic, and electrochemical diffractometry techniques can be used to determine the exact number of active sites and the real surface for any type of electrocatalyst. The key techniques[^87^, ^88^, ^89^] that hold a lot of promise as universal methods for determining the exact number of active sites include the operando IR, UV/Vis, Raman, X‐ray photoelectron, and X‐ray absorption spectroscopies, operando electrochemical atomic force, scanning tunnelling, and transmission electron microscopies, and operando X‐ray diffraction analysis. Few of the previously listed techniques can be directly used to determine the active sites, while the others can be used indirectly for the same.

#### Indirect Determination of Active Sites with Operando UV/Vis, Raman, and IR Spectroscopies

In electrocatalysis, operando UV/Vis, Raman, and IR spectroscopies[^90^, ^91^, ^92^, ^93^] were mainly used to elucidate the reaction mechanism of the catalytic reaction under study by following the spectroscopic characteristics of the intermediates involved in the reaction. The same strategy can also be used to determine the true electrochemical surface area and the corresponding exact number of electroactive sites as discussed below. UV/Vis/Raman/IR‐active probe molecules (can be neutral or ionic) capable of adsorbing on the electrode surface at an applied potential can be introduced into the electrolyte. The corresponding absorption/intensity/transmittance values of free probe molecules in the electrolyte at OCV and at operando conditions can become handy in determining the concentration of probe molecules adsorbed on the electrode surface. Alternatively, diffuse reflectance techniques in UV/Vis and IR spectroscopies can also be used to directly quantify the concentration of adsorbed probe molecules on the electrode surface. From this concentration, one can easily back‐calculate the real surface area and the number of active sites, provided that the lattice parameters are known. Though this method appears to be similar to the *C*_dl_ method, it is still advantageous, as it avoids the weak point of the *C*_dl_ method (i.e., adopting *C*_s_ values from other sources to calculate ECSA). However, since the absorption and transmittance of such probe molecules can be followed (with operando UV/Vis/Raman/IR spectroscopies) only within a narrow concentration range, high‐surface‐area electrodes may not be studied using these methods. Other than that, this method can be universally applicable to both metallic and metal‐free catalysts having more than one component.

#### Direct Quantification of Metallic Active Sites Using Operando Mössbauer, X‐ray Photoelectron, and X‐ray Absorption Spectroscopies

Mössbauer, X‐ray photoelectron (XP), and X‐ray absorption (XA) spectroscopies help us to find the oxidation states of the metal centers on a catalytic surface.[^94^, ^95^, ^96^, ^97^, ^98^, ^99^, ^100^] Among them, Mössbauer spectroscopy is limited to only Mössbauer‐active metals. Of several metals that can be studied with Mössbauer spectroscopy, Fe and Sn have been extensively used in oxygen electrocatalysis and CO_2_ electroreduction. Hence, it was obvious to use this technique in operando mode for tracking the changes in the oxidation state of Fe‐ and Sn‐containing electrocatalysts and photoelectrocatalysts. Similarly, the recently developed and more powerful ambient‐pressure XP spectroscopy is colossally beneficial in tracking the oxidation states of catalytic sites under catalytic turnover conditions. Alternatively, the intensity values obtained by these techniques for each element in a multimetallic catalyst can be used to quantify the catalytic sites if they are normalized by the work function of instrument. Provided that one has lattice parameters obtained from other characterizations, the calculation of the exact number of active sites and the real surface area under operando conditions can easily be done. Since the peak positions of different metals are sufficiently separated from one another in XP spectroscopy, it can easily be used to determine active sites of multimetallic catalytic systems, which is mostly impossible with other widely used methods. On the other hand, the operando XA spectroscopy can provide the more detailed information that one needs to calculate the exact number of active sites and the real surface area. The extended X‐ray absorption fine structure (EXAFS) spectroscopy and X‐ray absorption near edge structure (XANES) spectroscopy components of XA spectroscopy do provide precise information on the local environment around the catalytic site, coordination number, bond length, and oxidation state. From this information, it is even easier to calculate the number of active sites. Though these methods appear to be superior, they too have a serious concern. The high‐energy X‐rays in XP and XA spectroscopies and very high energy γ‐rays in Mössbauer spectroscopy can penetrate well below the surface of the catalyst and could provide misleading information on the amount of metal sites participating. Hence, only an atomically thin layer of a multimetallic catalytic system can be studied (ideally) using these techniques. Even though this is a serious shortcoming, there is no other single method for determining the exact number of active sites in a thin layer of a multimetallic catalyst (especially when it is amorphous). Moreover, owing to the recent huge interest in single‐atom catalysts (SAC)^[7]^ and noble‐metal dilution,[^52^, ^101^] there is a very high chance for the evolution of such amorphous multimetallic thin layer catalysts which will require operando Mössbauer, XP, and XA spectroscopies for the determination of the exact number of active sites.

#### Operando Microscopic Techniques for the Determination of the Number of Active Sites

Microscopic techniques such as AFM and STM under operando conditions can be the best tools to study and map the surface structure of a catalyst.[^102^, ^103^, ^104^, ^105^] AFM, which generally operates well in air, poses a few practical difficulties when used in an electrolyte (i.e., at the interface between a solid electrode and a liquid electrolyte). However, the recent advancements made in cell and AFM probe design have made it easier. The topographical information obtained through electrochemical AFM (EC‐AFM) under operando conditions is generally used to follow the structural changes occurring during an electrocatalytic reaction. However, if the area of analysis is extended to a sufficiently larger area until a repeating pattern (if any) is found, EC‐AFM can safely be used to extrapolate and determine the real surface area and the number of active sites. In this aspect, electrochemical STM (EC‐STM) is even more advantageous as it offers atomic precision. Hence, the surface mapping, real surface area calculation, and determination of exact number of active sites would be easy and straightforward with these techniques albeit time consuming. However, these methods cannot be used to study gas evolution reactions as the gas bubbles will severely affect the STM and AFM probes. Also, the best results can be obtained only for flat, rigid, and smooth electrodes. This limits application of STM and AFM to the study of 3D electrodes and electrocatalysts containing contact‐sensitive ligands such as porphyrins, phthalocyanines, and DNA. Apart from this, scanning electrochemical microscopy (SECM) is also a promising tool and perhaps, even better than EC‐AFM and EC‐STM. The major issues with this method is that there is no well‐characterized redox probes and the complexities associated with making and handling ultra‐microelectrodes (UME). Besides, like EC‐AFM and EC‐STM, this method also cannot be used for gas‐evolution reactions. Other techniques such as TEM and XRD under operando conditions can also give coessential information on the surface structure of the catalyst under catalytic turnover conditions that can be used for the determination of real surface area and the number active sites.[^106^, ^107^, ^108^] Based on this discussion, we believe that operando UV/Vis, Raman, and IR spectroscopy can be used universally for all types of catalysts (metallic, non‐metallic, multimetallic, flat and smooth, 3D electrodes, catalysts with touch‐sensitive ligands, etc.) but only for smaller electrodes, because for many probe molecules the concentration range for having a linear concentration–absorption/transmittance relationship is very narrow. On the other hand, if the electrode is flat, smooth, and atomically thin, all the other operando techniques proposed here may be used with no restrictions on the size of electrodes. That stated, we believe that these recently evolved in situ and operando methods may indeed be the universal ways we all have been anticipating for determining the real surface area and the exact number of active sites.

### Need for Normalizing the Current Responses with FE

While we strongly advocate the preferential use of TOF for accurately reflecting intrinsic activity, the significance of FE and its influence on accuracy of TOF determined ought not to be omitted.^[16]^ It is now made clearer that the precise determination of the exact number of active sites catalyzing the reaction is essential for an accurate TOF determination. Similarly, the FE of the catalyst is also crucial to ensure the accuracy of the TOF calculated. Most energy conversion electrocatalytic reactions are carried out in multiple steps, leading to different products with different selectivity. In many of them, the catalyst itself ought to be involved in a cycle of continuous oxidation and reduction. Hence, there is a fair chance that the FE could significantly be lower than the ideal value of 100 %. The only electrocatalytic reaction that is known to have 100 % efficiency for most of the catalysts studied is HER. In the case of OER, efficiency usually ranges from 85 to 96 % because a considerable amount of applied charge is consumed in the self‐redox reaction of the catalyst. The same is also true for ORR. On the other hand, the two‐electron water oxidation reaction that produces H_2_O_2_ usually shows poor FE in the range of 40–75 % because of the competitive OER occurring simultaneously. Reduction of CO_2_ and N_2_ also suffer from very low FE.

**Figure 8:**
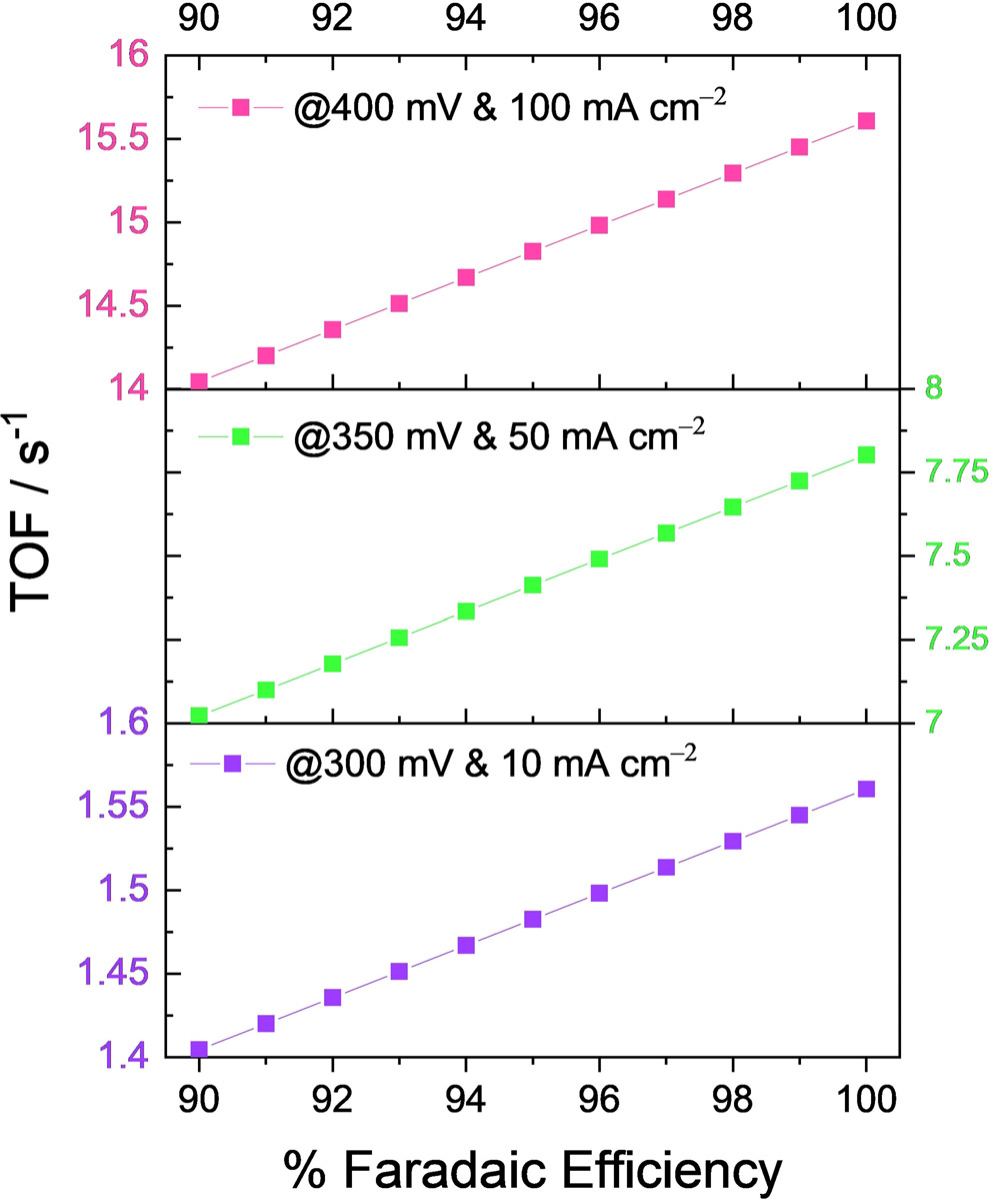
Effect of FE on the TOF determination of a putative OER electrocatalyst presumed to be delivering 10, 50, and 100 mA cm^−2^ at overpotentials 300, 350, and 400 mV, respectively with 1×10^16^ electrocatalytically active sites.

In cases such as these, if one uses whatever the current response that was obtained for TOF calculation without taking FE into consideration, the calculated TOF will be will deviate far from the true value. Figure 8 shows how significantly a small change in the FE can distort the TOF of a putative OER electrocatalyst. The presumed conditions are: it delivers 10, 50, and 100 mA cm^−2^ at the overpotentials of 300, 350, and 400 mV, respectively, and the number of sites participating in OER is 1×10^16^. To show the effect of even a small change of FE in the TOF calculations, TOF values at 300, 350, and 400 mV of this putative electrocatalyst were calculated using Equation (1) for different FE values ranging from 90 to 100 %. From Figure 8, it is evident how significant FE is in the precise determination of the TOF of an electrocatalyst. It also can be noticed that when the catalyst perform at high rates (i.e., at higher current densities which is usually the case with the commercial electrolysers), the influence of even a small change in the FE could significantly alter the calculated TOF values, as TOF and FE are proportional to one another. Hence, we strongly recommend the FE normalization of a current response, which was normalized already by the true/real electrochemical surface area, before using it for TOF determination.

### Recommended Steps for (Relatively) Accurate TOF Calculation Using Widely Used Methods

From the above discussion, it is clear that there is currently no method that can be both accurate and universally applicable to all kinds of electrocatalysts to determine the exact number of active sites participating in the reaction being catalyzed. Still, material‐specific methods such as UPD, stripping voltammetry, and redox‐peak integration offer us precise paths for many known catalysts. On the other hand, assuming 100 % participation of all the sites in the loaded catalyst offers universal applicability but demeans the purpose of accurate TOF determination.

**Scheme 1:**
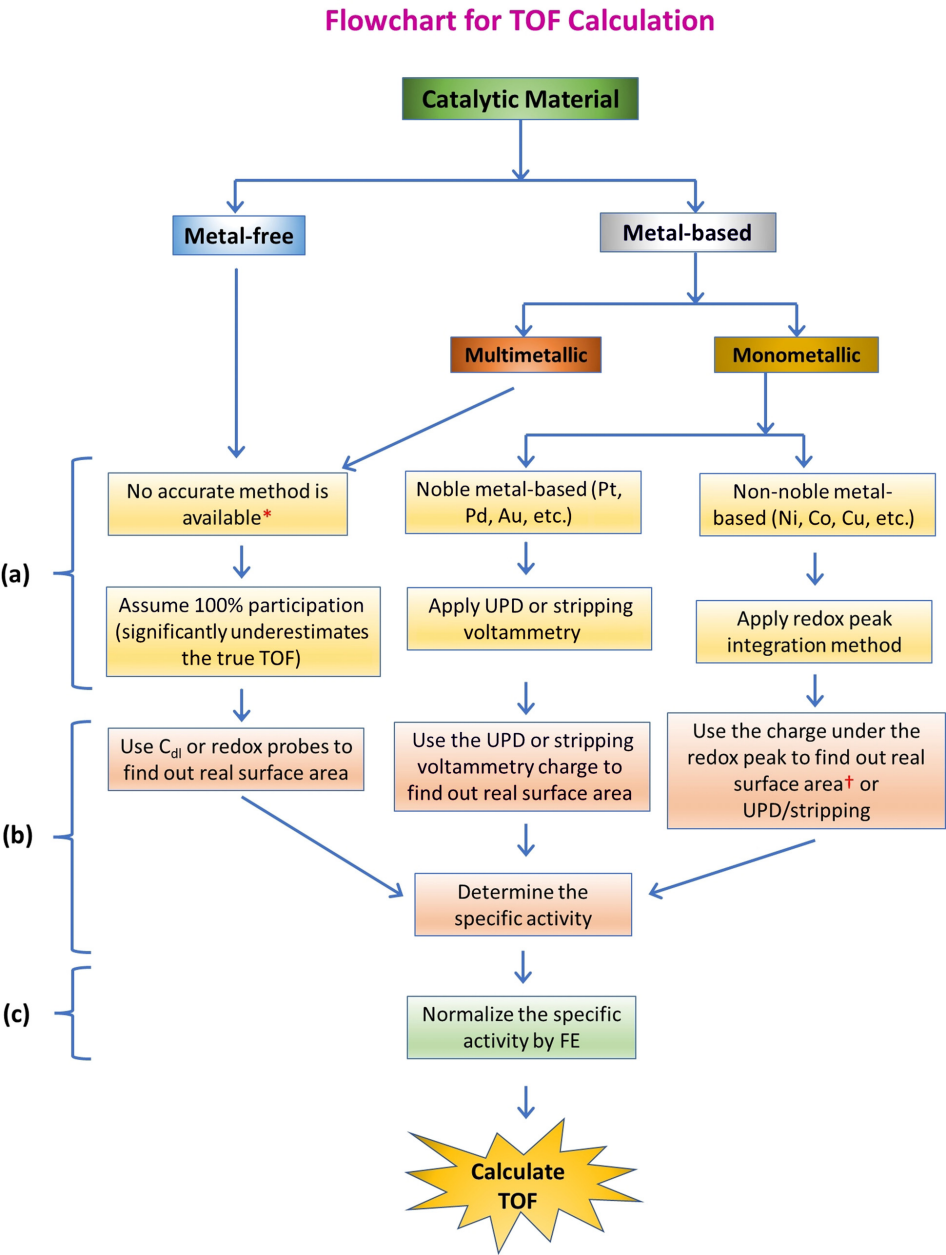
Flowchart showing the steps required to calculate a relatively accurate TOF for different electrocatalytic materials used in energy conversion electrocatalysis. a) The step determining the exact number of active sites participating in the catalysis, b) the step excluding errors imparted by other ways of current normalization (with geometrical area, mass, etc.), and c) the step excluding errors for catalysts with FE <100 %. [*] Electrochemical surface area obtained dividing *C*_dl_ by *C*_s_ and the other futuristic methods discussed earlier can be used only when lattice parameters are known. [†] Requires lattice parameters.

After calculating the exact number of active sites, one should focus on normalizing the current responses by the true/electrochemical surface area to get the specific activity. Most importantly, the specific activity obtained must be normalized for FE as well before using it for the TOF determination. Scheme 1 illustrates the systematic way in which TOF should be calculated in terms of both process and methods. It is evident that there are no accurate ways of determining the exact number of active sites, except in monometallic catalysts. For multimetallic and metal‐free catalysts, besides assuming 100 % participation of all the sites, the *C*_dl_ values can be used to back‐calculate the electrochemical surface area (by dividing the *C*_dl_ by *C*_s_), which in turn can be converted into the number of moles of active sites when the lattice parameters are known and the catalyst powder consists of single crystals of the same size and shape. All these requirements are rarely met for many catalysts. Though *C*_dl_ and *C*_s_ can be found and the lattice parameters could be obtained from diffraction studies, for most known catalytic materials it is next to impossible to achieve homogenous size and shape with single crystallinity. To sum up, we recommend the use of material‐specific well‐established methods that are known to reveal the exact number of active sites though they could be time‐consuming. Opting for the easier approach of assuming 100 % participation is not an accurate method to determine the exact number of active sites. When the catalyst is single crystalline and the lattice parameters are available, electrochemical surface area can be used to calculate. In the end, one should always remember to normalize the current response by the real/electrochemical surface area (i.e., finding specific activity) and FE before using it for TOF calculation. Thus, one can ensure that the TOF determined is more accurate.

Apart from this, the influence of substrates/supports/carriers such as metal foams, metal foils, CNT, graphene, carbon cloth (CC), carbon fiber paper (CFP), etc. should not be underestimated in an (relatively) accurate determination of the TOF of nanostructured catalysts loaded onto them. Though the activity of these substrate materials is usually not so high, they can notably alter the TOF since we give importance to the tinier possible changes in the FE as well in order to have a more accurate TOF. We propose two independent ways of overcoming this issue. The first is ensuring that the loading is sufficiently high so as to have 100 % surface coverage with metals foils, metal foams, CC, and CFP while being vigilant not to increase the resistance of the electrode and lower the mass activity. When we have 100 % surface coverage, the chances for the substrates getting exposed to the electrolyte and contributing to the catalysis is nearly zero. However, upon prolonged operation, increased wetting and the dissolution of the catalyst layer may expose the substrate and can hinder accurate TOF determination. Hence, a more rational method would be better. For that, we propose determining the TOF values of both the substrate and the catalyst‐loaded substrates separately and subtracting the TOF of the former from the latter. In this way, we don't have to have 100 % surface coverage or suffer from increased electrode resistance and lowered mass activity. For materials that are loaded on carriers such as CNTs having high specific surface area (SSA), it would be better to determine the TOF of the catalyst of the same mass on a different substrate with a smooth surface to avoid complications that may arise due to the concentration gradients and the formation of gas bubbles that mask sites deep in the pores. In cases where the loading of catalysts onto such high SSA carriers is the reason for the enhanced activity, determination of an accurate TOF can be cumbersome. In such a scenario, one may opt to study the interface at low currents to avoid the aforementioned complications. For other substrates, the inability of finding out the exact number of active sites may prohibit this method from being applied. For metal foils and foams, the redox‐peak integration method discussed in this work can be used, whereas the *C*_dl_ method can be used for carbon‐based substrates. In either case, the determination of an accurate TOF (intrinsic activity) is the objective and one can facilitate the design and redesign of electrocatalysts and electrocatalyst‐loaded electrodes for achieving better apparent activity with the same.

## Summary and Outlook

Energy conversion electrocatalysis (both fuel‐forming and fuel‐consuming) has been at the apex of applied electrochemistry research in the past few decades because electrochemistry offers several advantages for many industrial‐level processes developed earlier for accessing value‐added fuels/chemicals. As a result, researchers from various academic backgrounds have come together to study various aspects ranging from catalyst design, electrolyte engineering, and cell design to life cycle assessment. Of all of these topics, designing electrocatalysts and screening them for energy conversion electrocatalysis is the most active area in this field. In general, of the three targeted evaluation parameters (activity, selectivity, and stability), activity is determined mainly by measuring the current density delivered at a fixed overpotential or overpotential required for a defined benchmarking current density. These overpotentials never reflect the intrinsic activity but the rather apparent activity of many catalysts, besides underestimating or overestimating the activity because of the poor normalization conventions followed. Normalization methods such as using geometrical surface area, BET surface area, mass, etc. profoundly increase the misinterpretation of the apparent activity. Hence, for reporting the intrinsic activity of an electrocatalyst, an additional reliable marker is needed besides the apparent activity markers that are widely used these days. For this, TOF is an impeccable option.

In this Viewpoint, we stress the necessity of properly reporting TOF to reflect the intrinsic activity of an electrocatalyst. Unlike the overpotential reported at a benchmarking current density that serves basically an apparent activity marker, TOF gives us direct information on the rate of consumption of a fuel or the production of the desired fuel in an energy conversion reaction. All the apparent activity markers used currently are greatly flawed owing to parasitic reactions, thermodynamically and kinetically competing reactions, and the addition of capacitive current to the catalytic current. This implies that one cannot determine the intrinsic activity of an electrocatalyst just by relying on these overpotentials. Hence, a properly determined TOF reported in addition is the solution.

Though the TOF has been included in a significant number of reports published recently, most of them are not properly calculated. The main reason is that the determination of TOF for an electrocatalyst requires the knowledge of the exact number of active sites participating, real/electrochemical surface area, and FE, which are not easy to find. Real/electrochemical surface area and FE can be found relatively easily, but not the exact number of active sites participating in the reaction. From our experience, we unified and stream‐lined the way of calculating a relatively accurate TOF for all sort of catalytic materials and for all electrocatalytic energy conversion reaction (Scheme 1) and we also stressed why a properly calculated TOF should be reported as an intrinsic activity marker along with all the other commonly reported apparent activity markers such as overpotentials and *j*_0_. However, use of material‐specific techniques for monometallic catalysts and the unavailability of an accurate method of calculating exact number of active sites for multimetallic and metal‐free electrocatalysts still prohibit the widespread use of TOF as a widely‐adopted intrinsic activity marker. Developing universal electroanalytical methods for finding out the exact number of active sites in all type of electrocatalysts is the only way to achieve this goal. To do this, the opinions we provided on the use of in situ and *operando* electrochemical microscopic and spectroscopic techniques maybe the ways we are looking. Regardless of whether such developments occur in the future or not, we ought to start practicing the use of a properly determined TOF for reflecting the intrinsic activities in all electrocatalysis reports for the sake of its reliability and straightforwardness alongside the commonly used apparent activity markers (i.e., the overpotentials and *j*_0_).

## Conflict of interest

The authors declare no conflict of interest.
